# Dual-function perovskite light-emitting/sensing devices for optical interactive display

**DOI:** 10.1038/s41377-022-01036-8

**Published:** 2022-11-22

**Authors:** Songman Ju, Yangbin Zhu, Hailong Hu, Yang Liu, Zhongwei Xu, Jinping Zheng, Chaomin Mao, Yongshen Yu, Kaiyu Yang, Lihua Lin, Tailiang Guo, Fushan Li

**Affiliations:** 1 Institute of Optoelectronic Technology, Fuzhou University, Fuzhou, 350116 China; 2 Fujian Science & Technology Innovation Laboratory for Optoelectronic Information of China, Fuzhou, 350116 China; 3 School of Intelligent Manufacturing and Electronic Engineering, Wenzhou University of Technology, Wenzhou, 325035 China; 4 The Straits Institute of Flexible Electronics (SIFE, Future Technologies), Fujian Normal University, Fuzhou, 350117 China

**Keywords:** Inorganic LEDs, Quantum dots

## Abstract

Interactive display devices integrating multiple functions have become a development trend of display technology. The excellent luminescence properties of perovskite quantum dots (PQDs) make it an ideal luminescent material for the next generation of wide-color gamut displays. Here we design and fabricate dual-function light-sensing/displaying light-emitting devices based on PQDs. The devices can display information as an output port, and simultaneously sense outside light signals as an input port and modulate the display information in a non-contact mode. The dual functions were attributed to the device designs: (1) the hole transport layer in the devices also acts as the light-sensing layer to absorb outside light signals; (2) the introduced hole trapping layer interface can trap holes originating from the light-sensing layer, and thus tune the charge transport properties and the light-emitting intensities. The sensing and display behavior of the device can be further modulated by light signals with different time and space information. This fusion of sensing and display functions has broad prospects in non-contact interactive screens and communication ports.

## Abstract (web summary)

Schematic diagrams of SD-PQLED based on light interaction layer and light-emitting layer and proof-of-concept diagram of the light interactive display by a four-pixel SD-PQLEDs.

## Graphical abstract

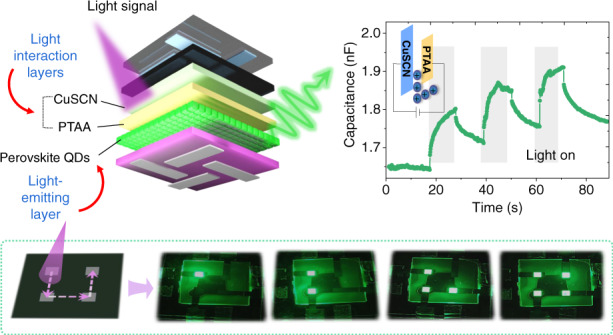

## Introduction

With the increasing demand for multi-functional and intelligent electronic equipment^1,2^, interactive displays have promising application prospects in many scenarios in real life. For example, a display panel with dual functions of sensing and display can provide real-time communication, allowing the device to sense outside signals and simultaneously display information. Such device endows light interaction between users and devices, devices and devices, and meets the multi-functionalization of a single device. Dual-function devices usually have different functions under forward and reverse bias working conditions, and further circuit design is required to realize the two functions at the same time. Therefore, it is particularly important to realize dual-function applications of the device under unidirectional working conditions without complicated circuit design. Traditional interaction methods are usually based on hardware tools (such as remote control, keyboard, mouse, handle or based on touch, gestures, somatosensory, etc., which are severely limited by distance, space, accuracy and scenes). Using light as the medium of human-computer interaction, accurate, remote, and large-scale interaction of display devices can be performed in non-contact mode, and it can be applied in many scenarios, such as game, drive, public display, medical and intelligent home, etc.

Since the first report of quantum dot light-emitting diodes (QLEDs) in 1994^3^, all kinds of quantum dots (QDs) candidate materials, including CdS^4–6^, CdSe^7–10^, InP^11–13^, etc., have been reported as potential luminescent materials for QLEDs. However, these QDs require a complicated core-shell synthesizing process, inert gas environment, and high synthesizing temperature^14–17^, posing a challenge for large-scale industrialization. Perovskite quantum dots (PQDs), due to its high defect tolerance, adjustable emission peak, narrow emission spectra and high photoluminescence quantum yields (PLQY) is considered as a kind of potential light-emitting QDs for QLEDs^18–23^. Moreover, in comparison with the above-mentioned conventional QDs, PQDs are compatible with simple room-temperature synthesizing methods in an ambient environment and exhibit excellent PLQY without elaborated core-shell heterojunctions, which have great prospects in the next generation of high-quality lighting and high-definition display^24–26^. Up to now, much effort has been focused on improving the light-emitting performance of perovskite QLEDs (PQLEDs), like external quantum efficiency (EQE) and device lifetime^27–31^. However, PQDs-based display with light-interactive function is still unexplored.

In this work, we propose a strategy to realize dual-function light-sensing/displaying PQLEDs (SD-PQLEDs). The devices not only can display information as an output port but also simultaneously sense outside light signals as an input port and modulate the display information accordingly. Moreover, by adjusting the temporal and spatial information of the outside light signal (such as light intensity and stimulation time), the light-emitting properties of the device can be tuned and even memory the signals. We demonstrated that light-interactive layer poly(bis(4-phenyl)(2,4,6-trimethylphenyl)amine) (PTAA) plays a key role in sensing outside light signal, and PTAA/copper thiocyanate (CuSCN) double-layer structure can trap holes from the PTAA layer and tune the charge transport properties. This SD-PQLED with dual functions of sensing and display provides an idea for the smart application of light-emitting devices, from non-contact interactive screens to display data communication.

## Results

**Fig. 1:**
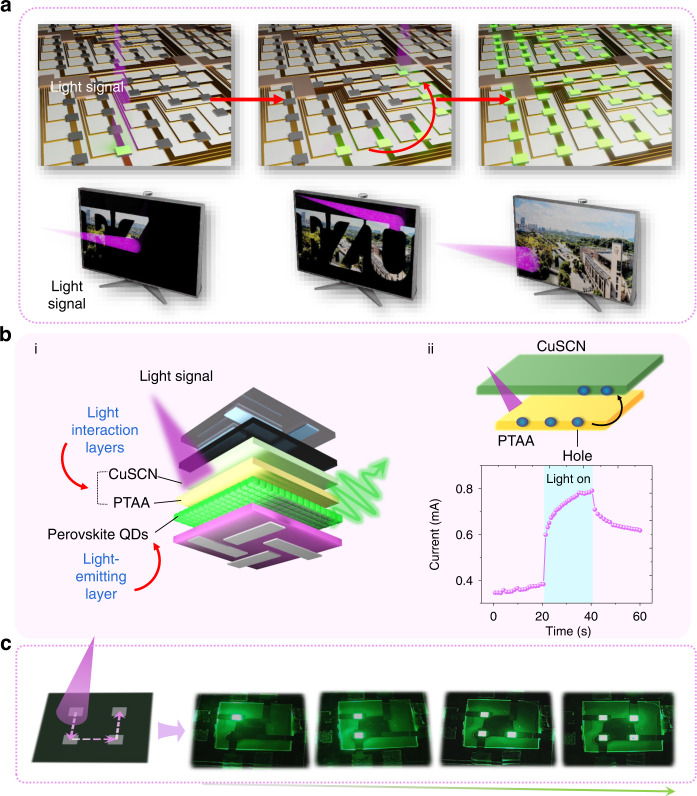
SD-PQLED design. **a** A conceptual diagram of a light-interactive display. The light pen illuminates the pixels of the display along the path depicted by the red arrow, and realizes the “light writing” on display. **b** i Schematic diagram of SD-PQLED based on light interaction layer and light-emitting layer; ii mechanism diagram of the optical interaction layer and the photocurrent of the device. **c** Proof-of-concept diagram of the light-interactive display by a four-pixel SD-PQLEDs

**Fig. 2:**
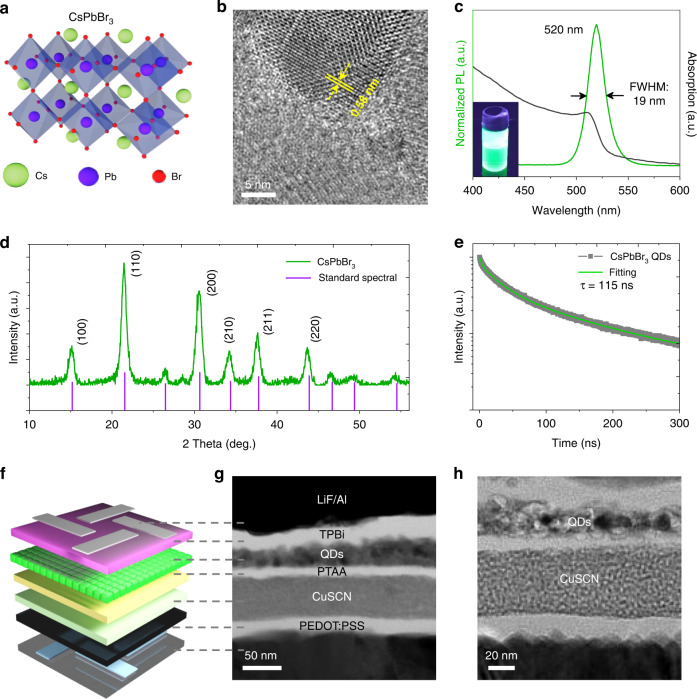
PQDs and devices characterization. **a** Schematic diagram of the lattice structure of CsPbBr_3_ PQDs. **b** HRTEM images of PQDs. **c** PL (green line) and absorption (black line) spectra of PQDs. The inset is a photoluminescence photo of PQDs ink. **d** XRD spectra of PQDs. **e** PL attenuation curve of PQDs ink. **f** Schematic of the SD-PQLED and **g**, **h** the pictures on the right are cross-sectional TEM images showing clear contrast between the multilayer devices

Figure 1a shows a conceptual diagram of an optical interactive display based on pixelated SD-PQLED. The user manipulates the smart display through a light pen without contact; when the pixel array of the display is illuminated by the light pen, the signal is then spatially sensed and analyzed by the pixel array, resulting in the “writing” action on display. The designed structure of SD-PQLED is schematically shown in Fig. 1b, which contains a PQDs light-emitting layer and a light-interactive interface based on the PTAA/CuSCN structure. PQDs act as the light-emitting material that enables the device to obtain electroluminescence (EL); the light-interactive interface plays a key role in light-interactive properties. In detail, PTAA, a widely-used hole transport layer in PQLED, can also sense ultraviolet (UV) light and generate electron-hole pairs. Followingly, the photo-generated holes can be captured by the CuSCN layer due to the PTAA/CuSCN interface barrier^32^, thereby generating a built-in electric field, which enhances the hole transport efficiency and increases the device current without changing driving voltages (Fig. 1b, ii)^33^. To demonstrate our device design with both sensing and display functions, we fabricated a 2 × 2 SD-PQLED array as a proof of concept (Fig. 1c). The light-emitting state of SD-PQLED can be lighted in sequence along with the light beam trajectory. The detailed fabrication process of the device is depicted in Fig. 2 and the experimental part.

Figure 2a shows the lattice structure of CsPbBr_3_ PQDs. According to the transmission electron microscope (TEM) images (Figure S1), the average particle size of the PQDs is 10 nm. The high-resolution TEM (HRTEM) image reveals a lattice spacing of 0.58 nm for PQDs, which is consistent with previous work (Fig. 2b)^25^. Fig. 2c shows the photoluminescence (PL) spectrum and ultraviolet-visible (UV–Vis) absorption spectrum of the CsPbBr_3_ PQDs. Under the UV light, the PQDs ink emits strong green light (the inset in Fig. 2c), with a PL peak of 520 nm and a full width at half maximum (FWHM) of 19 nm, which are beneficial for high-quality display. And the prepared PQDs ink shows high PLQY (95%), indicating good surface passivation and stability^34^. X-ray diffraction (XRD) shows broad peaks at 7.59°, 10.77°, 15.32°, 17.18°, 18.88° and 21.94°, corresponding to (100), (110), (200), (210), (211), and (220) of CsPbBr_3_ with the cubic crystal structure (PDF#54-0752), respectively (Fig. 2d). The PQDs film by spin coating shows dense and smooth morphology (Fig. S2). Fourier transform infrared spectroscopy (FTIR) characterizes the ligands of the PQDs. As shown in Fig. S3, there is no obvious absorption peak above 3000 cm^−1^, indicating no unsaturated C-H stretching vibration. The peaks at 2967 cm^−1^ and 2930 cm^−1^ are the antisymmetric stretching vibrations of CH_3_ and CH_2_ group, respectively. The symmetrical stretching vibration peak of the CH_3_ group is at 2863 cm^−1^, and the C–H bending vibration region is located at 1467 and 1378 cm^−1^. X-ray photoelectron spectroscopy (XPS) is used to detect the surface composition of PQDs, indicating the presence of Cs, Pb, C and Br elements (Fig. S4). We also measured time-resolved PL (TRPL) spectra to better understand the exciton recombination dynamics (Fig. 2e). The PL decay curve is fitted by a three-exponential function, indicating that the average lifetime of the CsPbBr_3_ PQDs ink is 115 ns. The short radiation lifetime indicates that the PQDs emit light through exciton recombination, which can be comprehended by the higher exciton binding energy of CsPbBr_3_ PQDs and the quantum confinement effect^35^.

We have prepared a device composed of indium tin oxide (ITO)/poly(3,4-ethylenedioxythiophene):poly(styrene sulfonate)(PEDOT:PSS)/PTAA/PQDs/1,3,5-tris(1-phenyl-1H-benzimidazol-2-yl)benzene (TPBi)/Lithium fluoride (LiF)/aluminum (Al), in which the TPBi, LiF and Al layers were prepared by thermal vacuum deposition, and the remaining layers were spin-coated on the prefabricated ITO in turn. Green CsPbBr_3_ PQDs as emitting layer, PTAA and TPBi as hole and electron transport layers, ITO and Al as anode and cathode of the device, respectively. Then we introduced a CuSCN layer between the PEDOT:PSS and PTAA as a hole extraction layer (Fig. 2f). And the cross-sectional TEM images of the device exhibit clear functional-layer boundaries, as shown in Fig. 2g–h.

**Fig. 3:**
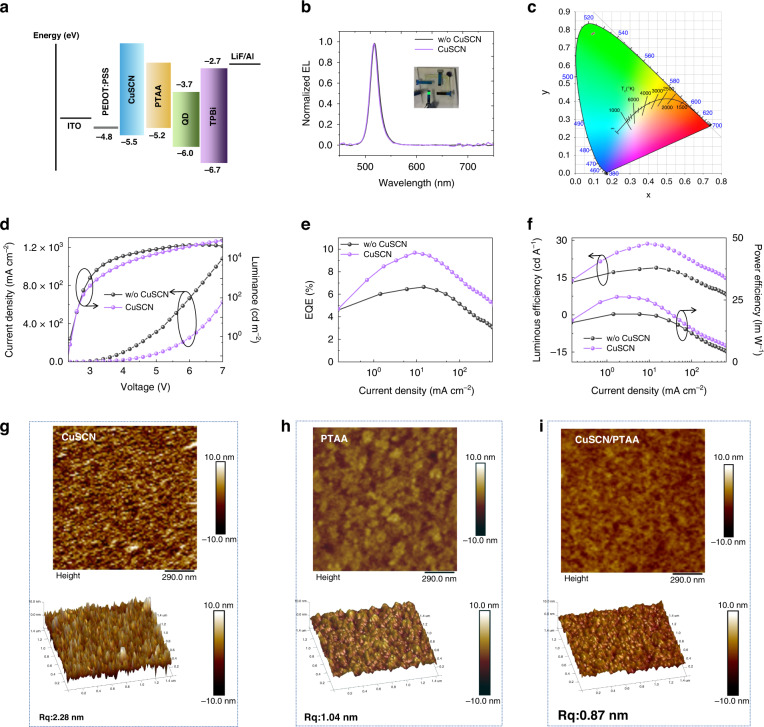
Light-emitting characteristics of PQLED with and without CuSCN layer. **a** Energy band diagram of SD-PQLED. **b** EL spectra of devices. The illustration is an electroluminescent photograph of the device. **c** Corresponding CIE coordinates of the devices. **d** J–V–L characteristics of the devices. **e** EQE of the devices as a function of current density. **f** LE and PE of the devices as a function of current density. AFM topography images of **g** CuSCN, **h** PTAA and **i** CuSCN/PTAA films deposited onto ITO substrates

Figure 3a shows the relative energy level diagram of each functional layer in the SD-PQLED. In Fig. 3b–f, we show the optical and electrical characteristics of the standard device (without CuSCN layer) and the SD-PQLED. Both devices present green EL peaks at 519 nm (Fig. 3b), which correspond to Commission Internationale de L’Eclairage (CIE) color coordinates of (0.10, 0.78) (Fig. 3c). Moreover, the EL spectra show a narrow FWHM of 19 nm, manifesting that the devices inherits the PL color purity of CsPbBr_3_ PQDs. The inset in Fig. 3b shows an EL photo of the SD-PQLED operating at 5 V, showing a bright green glow. Figure 3d shows current density (left ordinate)/luminance (right ordinate) versus voltage (J–V–L) characteristics of the standard device and SD-PQLED. As seen from the figure, the current density of PEDOT:PSS/CuSCN device is more than 100 times lower than that of PEDOT:PSS (electronic affinity ≈ 3.3 eV) device, which is due to the CuSCN (minimum conduction band (CBM) level = 2.0 eV) can act as an effective electron-blocking layer, making the device leakage shunt path less. It is worth noting that even with the introduction of a CuSCN layer, the turn-on voltage of the SD-PQLED remains at 2.4 V, the same as that of the standard device. Figure 3e and f compare the external quantum efficiency (EQE), luminous efficiency (LE) and power efficiency (PE) of the two devices. Table S1 summarizes some of the vital performance metrics obtained from these and Fig. 3d. After introducing the CuSCN layer, both the maximum brightness and efficiency are primarily enhanced in terms of light-emitting properties. The maximum brightness of SD-PQLED at 7 V reaches 75,898 cd m^−2^. The LE is 28.7 cd A^−1^ and a corresponding PE of 26.3 lm W^−1^, which are much higher than those of the standard device (19 cd A^−1^ and 19.3 lm W^−1^) (Fig. 3f). Therefore, the SD-PQLED exhibits a higher electro-optical conversion efficiency, with a peak EQE of 9.7% (Fig. 3e). Combined with the low maximum valence band (VBM) level of TPBi and the high CBM level of CuSCN, the excitons can be tightly confined within the emitting layer, resulting in the improved in the SD-PQLED efficiency. In terms of solution-processed multilayer PQLEDs, smooth and homogeneous functional films are of great importance for high-performance light-emitting devices. Herein, we used an atomic force microscope (AFM) to characterize the following three films: CuSCN, PTAA, and CuSCN/PTAA (Fig. 3g–i). It can be seen from Fig. 3i that the roughness of the CuSCN/PTAA double-layer film is 0.87 nm, which is smaller than that of the CuSCN (2.28 nm) and PTAA (1.04 nm) monolayers, indicating that the insertion of the CuSCN layer helps to improve the smoothness of the PTAA film rather than the roughness of the film. This film-forming advantage also positively affects the performance of the device. Figure S5 shows the histogram of the maximum EQE for devices with and without CuSCN. The EQEs of devices without CuSCN range from 5.95% to 8.25%, with an average of 7.09%. And the EQEs of devices with CuSCN range from 7.95% to 11.82%, with an average of 9.62%. It shows that both devices have good repeatability.

**Fig. 4:**
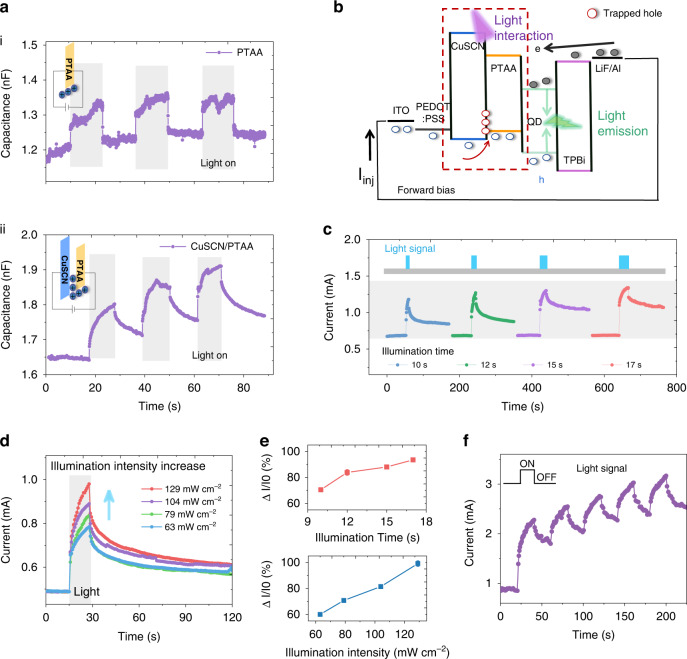
Light-interactive characteristics of SD-PQLED. **a** Curves of capacitance over time of PTAA (top) and CuSCN/PTAA (bottom) films with the light on and off. The insets show the working mechanism of the different functional layers. **b** The working mechanism diagram of SD-PQLED under UV stimulation. **c** Device current under different illumination time. **d** Current curves of the SD-PQLED under irradiation with different light intensities. **e** The change rate of current vs. illumination intensity (bottom) and illumination time (top). **f** Typical optical switching characteristics of the device under periodic UV light stimulation

In addition, the PQLED also possesses UV light-interactive properties, which we attribute to the CuSCN/PTAA bi-layer structure that can trap and release holes from the photo-sensing layer PTAA and tune the charge transport properties. Figure S6 shows the absorption spectra of the PEDOT:PSS, CuSCN, PTAA, PQDs and TPBi layers. We also tested the changes in the device current under different wavelengths of light stimulation (Fig. S7). The device shows obvious response to 365 nm light stimulation, weak response to 405 nm light stimulation, and almost no response to the light with wavelengths above 450 nm. According to the absorption band edge characteristics of these functional layers, we excluded the PEDOT:PSS, CuSCN, PQDs and TPBi layers, and considered the PTAA layer as the photosensitive layer of the device because of its absorption band edge between 365 nm and 450 nm. Stimulated by external light, the PTAA layer in the device absorbs the input photons and generates electron-hole pairs. We further tested the electrical response of the PTAA, PQDs, TPBi and PEDOT:PSS/CuSCN/PTAA films under UV light stimulation (Fig. S8). The results show that only the PTAA and PEDOT:PSS/CuSCN/PTAA films can generate significant photocurrent. In addition, we verified charge trapping behavior in the films through capacitance testing (Fig. 4a). We measured the capacitance values of the PTAA and CuSCN/PTAA films before and after light exposure. The results show that the capacitance value of the PTAA film increases significantly after being stimulated by light (Fig. 4a, i), and rapidly returns to the initial state after removing the light stimulus. The capacitance value of the CuSCN/PTAA film also greatly increase under light stimulation (Fig. 4a, ii), but does not fall back to the initial state after light removal. This charge retention behavior should be attributed to the existence of the CuSCN layer. As shown in Fig. 4b, when a light signal is applied to the device, a large amount of photo-generated holes originating from the CuSCN/PTAA films are injected into the PQDs, resulting in a significant increase in the device current and green emission intensity. And since the work function of the CuSCN film is 5.5 eV, which is higher than that of the PEDOT:PSS film (4.8 eV) and PTAA film (5.2 eV) (Fig. 3a), parts of the photon-induced holes are captured by this layer. After removing the external light, these holes are trapped in the CuSCN layer without the application of an external electric field^32^. Then, the charges retained in the CuSCN layer generate a built-in electric field that helps hole transport from the CuSCN layer to the PTAA layer, thereby improving the hole transport efficiency and conductance of the device^33,36^. During second light stimulation, both the current and brightness of the device increased significantly (Fig. S9b). To further reveal the influence of the CuSCN layer on photo-generated charge carriers, we carried out TRPL spectroscopy measurements on the PQDs deposited on glass substrate, PTAA and CuSCN/PTAA films, respectively (Fig. S10). Table S2 shows the average lifetimes of different functional layer films. It is not difficult to find that the lifetime of the PQDs deposited on the CuSCN/PTAA layer is lower than that of the PQDs deposited on the PTAA layer. This phenomenon of PL quenching further verifies that the insertion of the CuSCN layer facilitates carrier transfer at the PQDs/hole transport layer interface^37–40^.

The light-interactive properties of the SD-PQLED can be further modulated by the illumination time and intensity of UV light, which is important for sensor display. The current variation of the device under modulation of illumination time is depicted in Fig. 4c. When fixing the light signal intensity at 79 mW cm^−2^, and tuning the illumination time as 10, 12, 15 and 17 s, the current of the device increases continuously with the illumination time. When irradiated for 17 s, the device current increased by 95% from the initial value (Fig. 4e). We also investigate the effect of light intensity on device current (Fig. 4d). Under the same light stimulation time, as light signal intensity increased from 63 to 129 mW cm^−2^, the current increase rate of the device increased from 60% to 100% (Fig. 4e). This is because the illumination intensity reflects the spatial information of the optical signal, determines the amount of photo-generated carriers per unit time, and causes current variation in the device. When the optical signal was removed, all currents showed a slight attenuation instead of returning to their original levels, meaning the device could remember previous states of light-interactive and display them. We also characterized the process that the current of the device vary with the number of light stimuli. As can be seen from Fig. 4f that after the first and fifth light stimulation, the device current increases by 120% and 180% compared to the initial value, respectively. Next, we tested the performances of the device before and after UV stimulation. After UV stimulation, the EL spectrum of the device remains unchanged (Fig. S11a), the current and brightness of the device increase by 118% and 108% at 4 V, and 170% and 77% at 6 V, respectively (Fig. S11b, c). The EQE of the device varies little (Fig. S11d). Moreover, by applying reverse voltage to release the charges trapped by the CuSCN, we demonstrate that the remembered display states can be erased for the following optical writing processes (Fig. S12), and the corresponding schematic diagram is shown in Figure S13.

## Discussion

In summary, we have prepared light-emitting devices based on PQDs that can realize the dual-function applications of photosensitive/display in non-contact mode. Here, we introduce a hole extraction layer (CuSCN) to trap the photo-generated holes originating from the PTAA layer and adjust the charge transport characteristics. The sensing and display behavior of the device can be further modulated by optical signals of different temporal and spatial information. In consequence, the device achieve a maximum peak EQE of 9.7%, and the current and brightness increase by 118% and 108% respectively (at 4 V) after a single light stimulation, showing strong light interaction and display capabilities. The proposed photosensitive/display dual-function device can not only enhance the established display capability, but also integrate multiple functions into one. Due to the latest development of solution processing capabilities and ultra-high color purity multicolor PQDs, this type of display composed of a large number of SD-PQLEDs has broad prospects in non-contact interactive screens.

## Materials and methods

### Chemicals

All chemicals were obtained commercially and without further purification. Cesium carbonate (Cs_2_CO_3_, 99.99%), Formamidine acetate (FA(Ac), 99%), n-Octanoic acid (OTAc, 99%), Tetraoctylammonium bromide (TOAB, 98%), Didodecyldimethylammonium bromide (DDAB, 98%), Copper thiocyanate (CuSCN, 99%), Diethyl sulfide (97%), Ethylacetate (99.5%), n-octane (> 99%) were purchased from Aladdin. Lead bromide (PbBr_2_, 99.999%) and Chlorobenzene (≥99.5%) were purchased from Sigma-Aldrich. Toluene (≥99.5%) were purchased from Sinopharm Chemical Reagent Co., Ltd. PEDOT:PSS, PTAA and TPBi were purchased from Xi’an *p*-OLED Photoelectric Technology Co., LTD. LiF and Al were purchased from Zhongnuo New Material Technology Co., LTD.

### Synthesis and purification of CsPbBr_3_ PQDs

First, mixture of 1 mmol Cs_2_CO_3_ and 10 mL OTAc, and mixture of 2 mmol FA(Ac) and 10 mL OTAc were loaded into two sample bottles respectively, and stirred at room temperature for 30 min to prepare cesium precursor and formamidine precursor. 1 mmol PbBr_2_ and 2 mmol TOAB were dissolved in 10 mL toluene to prepare lead precursor. Second, 0.85 mL cesium precursor solution and 0.15 mL formamidine precursor solution were quickly added to 9 mL lead precursor solution, and magnetically stirred for 5 min in the atmosphere at room temperature. Then, 3 mL DDAB solution (10 mg mL^−1^ in toluene) was quickly added to the reactant to obtain CsPbBr_3_ PQDs crude solution. After vigorous stirring for 2 min, twice the volume of ethyl acetate was added to the crude solution. After centrifugation at 10,000 rpm for 10 min, the sediment was collected and dispersed in toluene. With additional ethyl acetate and further centrifugation, the sediment was dissolved in n-octane to obtain the final PQDs.

### Device fabrication

ITO-coated glass substrates were cleaned ultrasonically with acetone and isopropanol in sequence. Use flowing nitrogen gas to dry the substrates. PEDOT:PSS solution was filtered through a 0.22 µm filter and spin-coated on the substrate at 4000 rpm for 40 s, then baked at 120 °C for 20 min to obtain a flat film. CuSCN solution (25 mg mL^−1^ in diethyl sulfide) was spin-coated on the PEDOT:PSS film at 3000 rpm for 40 s and baked at 120 °C for 20 min. PTAA solution (5 mg mL^−1^ in chlorobenzene) was spin-coated on the PTAA film at 2000 rpm for 40 s and baked at 120 °C for 20 min. CsPbBr_3_ PQDs (in n-octane) were spin-coated on the previous film at 2000 rpm for 60 s and baked at 60 °C for 10 min. TPBi (40 nm), LiF (1 nm) and Al (100 nm) were deposited by a shadow mask using a thermal evaporation system. The light-emitting area of the device is 4 mm^2^, which is determined by the overlap of the ITO anode and the Al cathode.

### Characterization and device measurement

The microscopic morphologies of PQDs were characterized using TEM and HRTEM (TECNAI G2 F20). The XRD samples were prepared by dropping PQDs ink onto the silicon wafer, and the XRD patterns were obtained by testing with the X’Pert PRO diffractometer (PANalytical). The PL spectra were collected by using a fluorescence spectrophotometer (Hitachi F-4600). The TRPL spectrum was obtained by using a fluorescence lifetime measurement system (HORIBA Scientific). The morphology of the CsPbBr_3_ PQDs film was measured by using a fluorescence microscope (Olympus BX51M). The FTIR spectrum of PQDs was obtained by using a FTIR spectrometer (Nicolet Avatar 360). PQDs ink was dropped on a silicon wafer to prepare XPS samples. The XPS spectra were measured by using a X-ray photoelectron spectrometer (ESCALAB 250). The UV–Vis absorption spectra of PQDs inks and films with different functional layers were obtained with a UV/Vis/NIR spectrophotometer (Shimadzu UV-3600). The materials were spin-coated on the ITO-etched glass substrates, and then the roughness results of the films were evaluated by the atomic force microscope (Bruker Multimode 8). The EL spectra, L-J-V characteristics, LE, PE and EQE were measured in a glove box filled with N_2_ using Keithley 2400 light source, fiber integrating sphere and PMA-12 spectrometer. The optical response and capacitance characteristics of the devices were obtained using the semiconductor characterization system (Keithley 4200).

## Supplementary information

Supporting information

Video
