# Supplementary material for: Dual-function perovskite light-emitting/sensing devices for optical interactive display
Source: Light Sci Appl. 2022 Nov 22;11:331. doi: 10.1038/s41377-022-01036-8 (PMC9684532; doi:10.1038/s41377-022-01036-8)
Supplement: Supplementary file 1 — Supporting information [file 41377_2022_1036_MOESM1_ESM.docx]

**Supplementary Information for**

**Dual-Function Perovskite Light Emitting/Sensing Devices for Optical Interactive Display**

Songman Ju^1,2&^, Yangbin Zhu^3&^, Hailong Hu^1^, Yang Liu^4^, Zhongwei Xu^1^, Jinping Zheng^1^, Chaomin Mao^1^, Yongshen Yu^1^, Kaiyu Yang^1^, Lihua Lin^1^, Tailiang Guo^1,2^ and Fushan Li^1,2*^

*^1^Institute of Optoelectronic Technology, Fuzhou University, Fuzhou 350116, China*

*^2^Fujian Science & Technology Innovation Laboratory for Optoelectronic Information of China, Fuzhou 350116, China*

*^3^School of Intelligent Manufacturing and Electronic Engineering, Wenzhou University of Technology, Wenzhou 325035, China*

*^4^The Straits Institute of Flexible Electronics (SIFE, Future Technologies), Fujian Normal University, Fuzhou 350117, China*

^&^These authors contributed equally to this work.


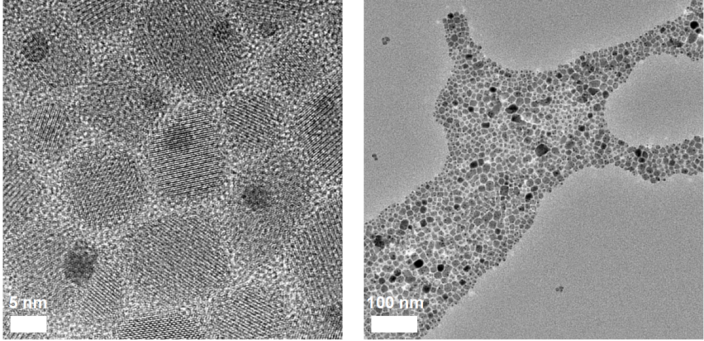


**Figure S1.** Transmission electron microscope (TEM) photographs of PQDs.


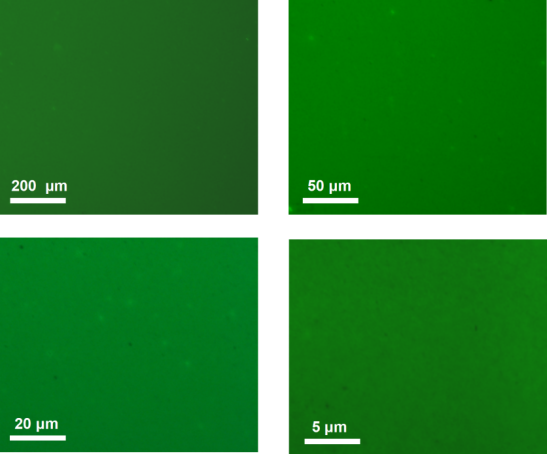


**Figure S2.** Fluorescence microscope photographs of PQDs films with different magnification.


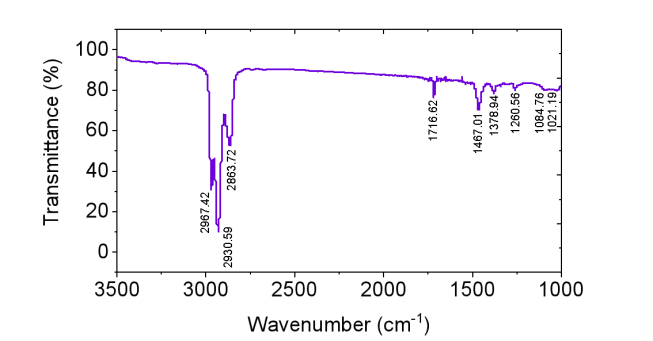


**Figure S3.** FTIR spectrum of PQDs.


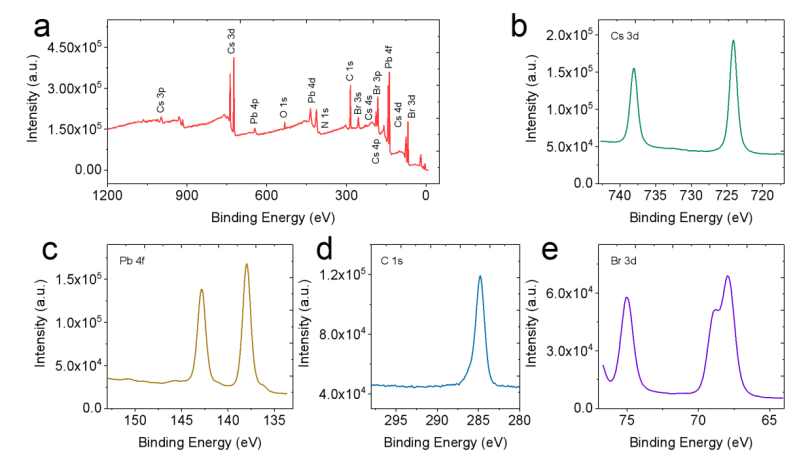


**Figure S4.** (a) XPS spectra of PQDs. (b-e) Cs 3d, Pb 4f, C 1s and Br 3d XPS spectra of PQDs.


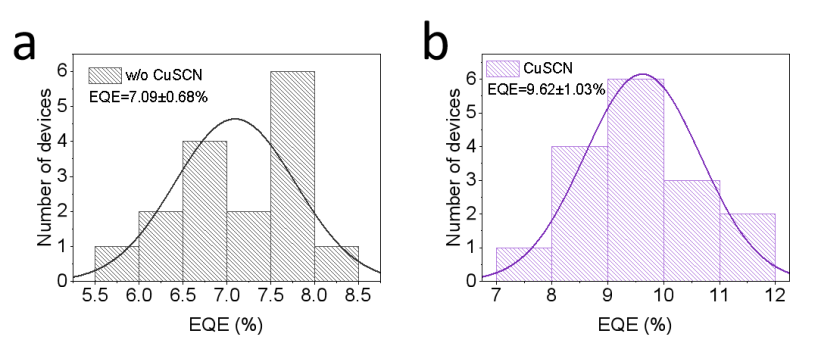


**Figure S5**. Statistical histograms of EQEs for (a) without and (b) with CuSCN devices.


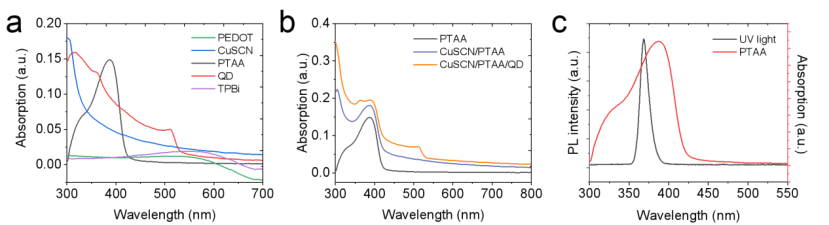


**Figure S6.** (a) Absorption curves of PEDOT:PSS, CuSCN, PTAA, PQDs and TPBi films. (b) For 365 nm UV light, the PTAA film exhibits strong absorption. (c) Absorption spectrum of PTAA film and PL spectrum of UV light.


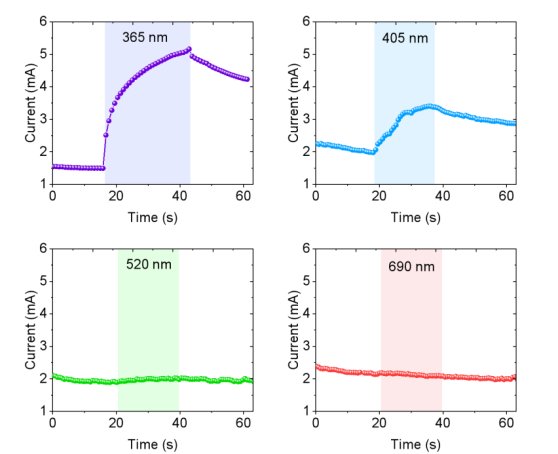


**Figure S7.** Changes in current of SD-PQLED under light stimulations with different wavelengths.


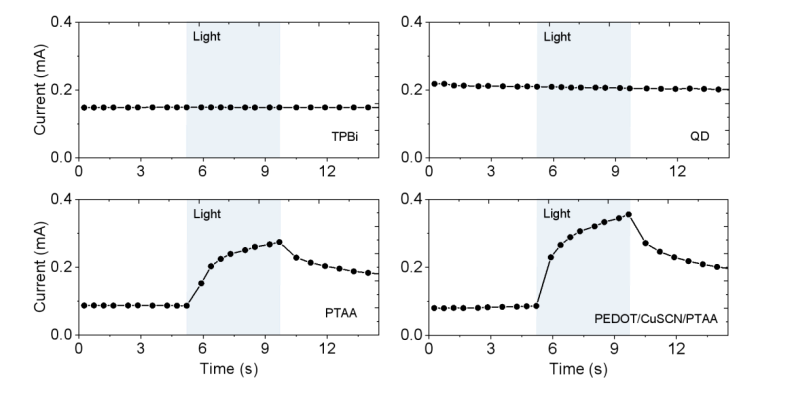


**Figure S8.** Changes in current of different functional layer films under UV light stimulation.


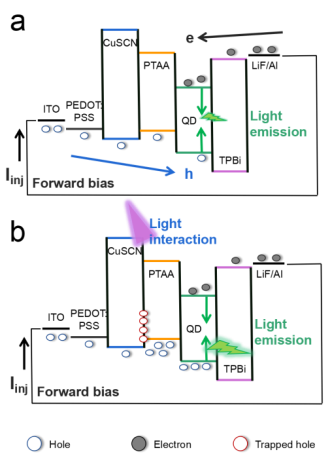


**Figure S9.** Working mechanism of SD-PQLED: (a) hole transport operation under voltage stimulation before illumination, (b) hole transport operation under second light stimulation.


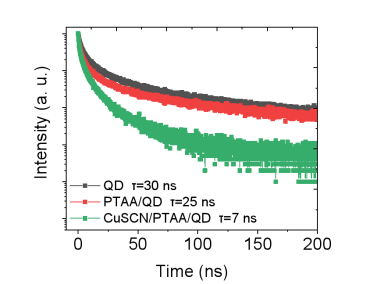


**Figure S10.** TRPL spectra for different functional layer films.


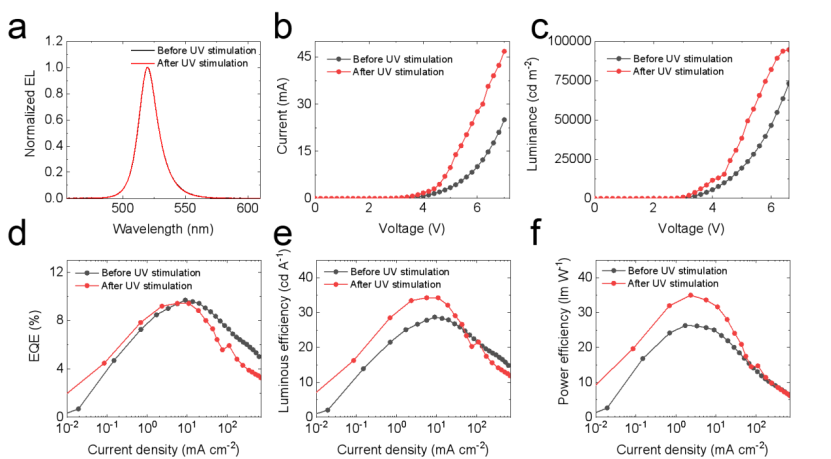


**Figure S11.** Performances of the SD-PQLED before and after UV stimulation.


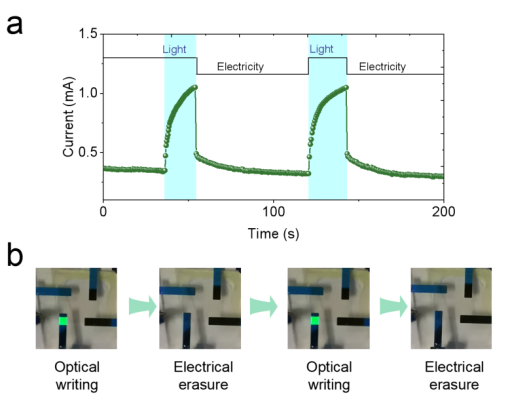


**Figure S12.** (a) Current changes in SD-PQLED under light and electrical stimulation. (b) Photos of device embodying optical writing and electrical erasure functions.


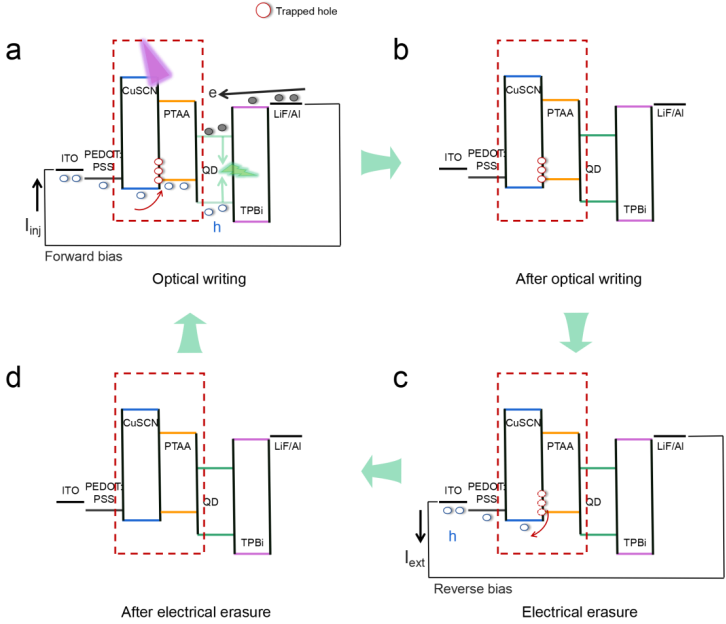


**Figure S13.** Schematic diagrams of optical writing and electrical erasure functions.

**Table S1. Summary of the performance of the devices with and without CuSCN.**

| Sample | EL peak [nm] | best luminance [cd m^-2^] | best EQE [%] | V_on_  [V] | best LE [cd A^-1^] | best PE [lm W^-1^] |
| --- | --- | --- | --- | --- | --- | --- |
| QLED | 519 | 55811 | 6.6 | 2.4 | 19.0 | 19.3 |
| CuSCN-QLED | 519 | 92918 | 9.7 | 2.4 | 28.7 | 26.3 |

**Table S2. Calculations of the average lifetimes (*T*_average_) of CsPbBr_3_ PQDs inks and different functional layer films as determined from the best fits to tri-exponential functions.**

| Sample | *T*_1_ (ns) | *T*_2_ (ns) | *T*_3_ (ns) | A_1_ | A_2_ | A_3_ | *T*_ave_ (ns) |
| --- | --- | --- | --- | --- | --- | --- | --- |
| QD ink | 3.48 | 28.59 | 138.33 | 1691 | 4268 | 3525 | 115 |
| QD film | 1.34 | 7.41 | 48.51 | 5148 | 3716 | 969 | 30 |
| PTAA/QD | 4.85 | 0.83 | 41.47 | 3454 | 5977 | 707 | 25 |
| CuSCN/QD | 0.27 | 3.14 | 16.51 | 9141 | 1047 | 370 | 9 |
| PTAA/CuSCN/QD | 0.60 | 3.33 | 14.78 | 6522 | 3429 | 786 | 7 |

The calculation details see Note S1.

**Note S1: Calculations of the average lifetimes (*T*_ave_).**

The PL decay curve can be well-fitted with the tri-exponential function:

$$decay\left(t\right)=\sum_{n=1}^{3} A_{n}e^{-t/T_{n}}$$

where A_1_, A_2_, and A_3_ are constants, *t* is time, and *T*_1_, *T*_2_, and *T*_3_ represent the decay lifetimes corresponding to the intrinsic exciton relaxation, the interaction between excitons and phonons, and the interaction between excitons and defects, respectively. The average lifetime (*T*_ave_) can be calculated as:

$$T_{ave}=\frac{\sum_{n=1}^{3} A_{n}T_{n}^{2}}{\sum_{n=1}^{3} A_{n}T_{n}}$$
